# Effect of Drying Methods on the Thermal and Mechanical Behavior of Bacterial Cellulose Aerogel

**DOI:** 10.3390/gels10070474

**Published:** 2024-07-18

**Authors:** Sebnem Sozcu, Jaroslava Frajova, Jakub Wiener, Mohanapriya Venkataraman, Blanka Tomkova, Jiri Militky

**Affiliations:** Department of Material Engineering, Faculty of Textile Engineering, Technical University of Liberec, 46117 Liberec, Czech Republic; jaroslava.frajova@tul.cz (J.F.); jakub.wiener@tul.cz (J.W.); blanka.tomkova@tul.cz (B.T.); jiri.militky@tul.cz (J.M.)

**Keywords:** bacterial cellulose hydrogel, lyophilization, thermal and mechanical behavior, structure, drying methods

## Abstract

Bacterial cellulose (BC) presents significant promise as a biomaterial, boasting unique qualities such as exceptional cellulose purity, robust mechanical strength, heightened crystalline structure, and biodegradability. Several studies have highlighted specific effects, such as the impact of dehydration/rehydration on BC tensile strength, the influence of polymer treatment methods on mechanical properties, the correlation between microorganism type, drying method, and Young’s modulus value, and the relationship between culture medium composition, pH, and crystallinity. Drying methods are crucial to the structure, performance, and application of BC films. Research findings indicate that the method used for drying can influence the mechanical properties of BC films, including parameters such as tensile strength, Young’s modulus, and water absorption capacity, as well as the micromorphology, crystallinity, and thermal characteristics of the material. Their versatility makes them potential biomaterials applicable in various fields, including thermal and acoustic insulation, owing to their distinct thermal and mechanical attributes. This review delves into the thermal and mechanical behavior of bacterial cellulose aerogels, which are profoundly impacted by their drying mechanism.

## 1. Introduction

In recent years, there has been a growing emphasis on the development of bio-based composites. Many studies have thoroughly explored different categories of bio-based materials, including cellulose and its derivatives, chitin-chitosan, polylactic acid, and polybutylene succinate. These materials are recognized for their potential environmental safety and non-toxicity, even when they reach the end-of-use stage [1,2,3]. Consequently, bio-based materials have seen widespread development, spanning from academic research to industrial adoption across various applications. These applications include infrastructure and building materials, automotive components, food packaging, as well as medical and pharmaceutical technologies. Moreover, the exploration of bio-based materials has centered on the concept of the bio-economy, which entails leveraging biotechnology-driven economic activities to establish industrial processes [4]. Cellulose is a widely available, eco-friendly material known for its mechanical strength, biocompatibility, hydrophilicity, and thermostability [5]. Traditionally used in energy, construction, fabrics, and paper production, technological advancements have expanded its applications to chemical-based materials. Research has investigated its use in pharmaceuticals, membranes and filtration, drug delivery, and as a stabilizer and emulsifier [6,7,8]. Additionally, cellulose has been advanced for paper-based technologies, such as bioactive paper for clinical diagnosis, environmental monitoring, food quality control, and biosensors. Innovations include gamma-irradiated paper and biohybrid cellulose paper with wet strength properties. However, producing high-purity cellulose requires removing lignin, hemicellulose, and pectin, a process that is pollution-prone and energy-intensive (about 1000 kWh/ton) with costly waste treatment [9,10]. Consequently, developing highly pure and environmentally friendly cellulose remains a key focus [11].

Aerogels are extremely porous open-cell solids with roughly 90% porosity produced by a supercritical drying method. Their relatively low thermal conductivity (from 0.013 W/m K) results in them being ideal thermal super-insulators. The low density (80–200 kg/m^3^) and complicated solid nanostructure, with mesopore widths ranging from 4 to 20 nm, reduce heat conduction and contribute to their high efficiency [12,13,14]. Aerogels can be made from a variety of resources, including inorganic compounds, synthetic polymers, and natural polymers [15,16,17], such as cellulose [18]. The initial component defines the particular kind of aerogel formed [19,20]. Cellulose aerogels are notable for their renewability, biocompatibility, and biodegradability, along with low density (0.5–350 kg/m^3^), high porosity (84.0–99.9%), and a large specific surface area. These characteristics make them highly promising materials for the 21st century. They also offer superior compressive strength (0.0052–16.67 MPa) and excellent biodegradability. Eco-friendly and versatile, cellulose aerogels are ideal for applications in adsorption and oil/water separation, heat separation, biomedical materials, and metal nanoparticle/metal oxide carriers and as precursors for carbon aerogels. Their high reusability reduces economic losses, and their natural decomposition prevents additional environmental harm [21,22].

Bacterial cellulose represents an alternative source of cellulose distinct from plant-derived cellulose. It is synthesized by various genera of bacteria, including *Acetobacter*, *Achromobacter*, *Agrobacterium*, and *Sarcina* [23]. It serves as a hydrogel due to its inherent purity and remarkable hydrophilicity, necessitating no additional treatment and preserving its original properties. These distinctive attributes of bacterial cellulose pave the way for numerous novel applications, as its properties can be tailored by adjusting the fermentation process. In comparison to plant cellulose, bacterial cellulose offers several advantages. Foremost among these is its exceptional purity, as it lacks the hemicelluloses and lignin found in plant cellulose, thereby reducing the need for extensive processing steps. Additionally, the structure of bacterial cellulose is superior, characterized by longer and finer fiber lengths that are approximately a hundred times smaller in proportion, contributing to its enhanced strength and fineness compared to plant cellulose [24]. The cellulose chains are bound together by both intra- and intermolecular hydrogen bonds, which gives BC its specific properties like exceptional purity, strong water retention, poor solubility, mechanical resilience, plasticity, biodegradability, biocompatibility, non-toxicity, and non-allergenicity [25]. Furthermore, its physiochemical qualities make it desirable for usage in both the textile and paper industries along with the other applications because of its multifunctionality. Its Young’s modulus is 20,000 mPa for sheets and 130,000 mPa for single fibers compared to plant cellulose, which is 25–200 mPa [26].

The properties of BC, such as degree of polymerization, crystallinity, and moisture absorption, can undergo alterations depending on various factors, including culturing conditions, the type of microorganism, and nutrients present in the growth medium. Several studies have highlighted specific effects, such as the impact of dehydration/rehydration on BC tensile strength [27]; the influence of polymer treatment methods on mechanical properties [28]; the correlation between microorganism type, drying method, and Young’s modulus value [29]; and the relationship between culture medium composition, pH, and crystallinity [30]. Furthermore, incorporating collagen between bacterial cellulose fibers has been shown to improve thermal stability and cytocompatibility through collagen fibrillogenesis, contributing to enhanced properties of BC-based materials [31]. Drying methods are crucial to the structure, performance, and application of BC films [32]. Research findings indicate that the method used for drying can influence the mechanical properties of bacterial cellulose (BC) films, including parameters such as tensile strength, Young’s modulus, and water absorption capacity, as well as the micromorphology, crystallinity, and thermal characteristics of the material [24,32,33,34]. This review examines the effects of various drying techniques on the thermal and mechanical properties of bacterial cellulose aerogels and their composites, highlighting their multifunctional applications and future potential. The review mostly encompasses studies from 2014 to 2024, focusing on different bacterial cellulose aerogels and comparing the impacts of various drying methods on their properties. Non-peer-reviewed articles were excluded from the analysis.

## 2. Production of Bacterial Cellulose Aerogel

**Figure 1:**
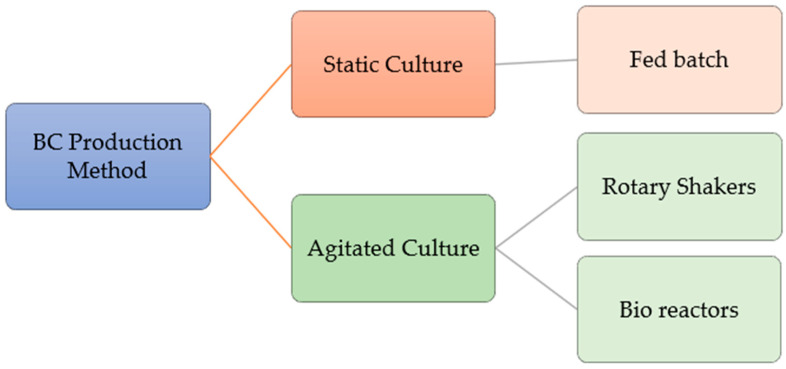
Categorization of production method of bacterial cellulose.

The cellulose produced by bacteria (BC) stands out from other cellulose sources for environmentally friendly applications. BC, unlike plant cellulose, is an extracellular polymer, which allows for high-purity extraction devoid of lignin and hemicellulose [35,36]. It has a high degree of polymerization and crystallinity, as well as a unique micro- or nano-sized fiber network, which increases its surface-to-volume ratio [37]. Bacterial cellulose (BC) production is mostly dependent on the nutritional makeup of the production medium, particularly carbon sources. Although many carbon sources and culture mediums have been investigated, the Hestrin–Schramm (HS) medium is the most often utilized [38,39]. However, its high cost, which accounts for 30% of overall production expenses, presents a significant barrier to commercial-scale fabrication [39,40]. Using agro-industrial waste for sustainable BC production is a desirable and cost-effective option [41,42]. Additionally, the selection of a BC-producing bacterium is critical [43], and cellulose production by bacteria has been observed across various species. Among the bacterial strains identified, the majority belong to Gram-negative species such as *Acetobacter*, *Azotobacter*, *Rhizobium*, *Pseudomonas*, *Salmonella*, *Agrobacterium*, *Aerobacter*, *Achromobacter*, and *Alcaligenes*. Additionally, Gram-positive species like *Sarcina ventriculi*, *Salmonella*, and *Escherichia* also demonstrate the capability to produce cellulose. Among the various bacterial strains identified, *A. xylinum* is recognized as one of the most promising strains for cellulose production due to its large production capacity, made possible by many copies of BC synthesis genes in its genome [38,44]. It has been documented that under optimal conditions, bacteria can convert nearly 50% of the carbon source provided in the medium into cellulose, forming a pellicle. Specifically, *A. xylinum* is a Gram-negative aerobic strain characterized by its rod-shaped morphology. This bacterium produces cellulose through its primary metabolic activity, forming interwoven ribbons. A notable advantage of *A. xylinum* is its ability to produce cellulose from various carbon sources. BC finds application in diverse fields, necessitating the optimization of its production for economic viability. Substantial research has explored optimal growth conditions to achieve high BC yields across different media, cultivation techniques, and environmental factors, including agitation levels, carbon and nitrogen sources, incubation duration, and medium volume [45,46]. Additional factors such as microorganism type, pH levels, and oxygen supply have been thoroughly investigated as they directly influence the characteristics of BC. Such studies are essential for comprehending how growth conditions affect the morphology and properties of BC [11]. As the cellulose structure and its physical and mechanical characteristics are purely affected by the cultivation process of bacterial cellulose, the methods for producing bacterial cellulose are critical parameters. Generally, cellulose production by bacteria occurs under two distinct conditions: (i) static conditions and (ii) agitated conditions (Figure 1 depicts the methods). Under static conditions, the bacterial culture and medium are left undisturbed in an aerated dark environment until fermentation is complete. In the agitated culture method, continuous stirring is maintained throughout the fermentation period. In the former case, cellulose pellicle forms as a continuous mat-like structure, while in the latter case, although cellulose growth is faster, there occurs the formation of round cellulose balls [47].

### 2.1. Static Culture

**Figure 2:**
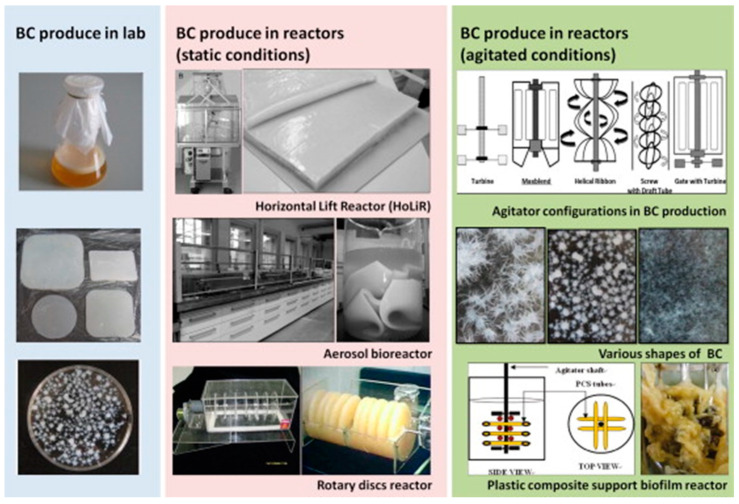
BC generated in static and agitated/shaking environments (permission was approved by Elsevier, Food Hydrocolloids, under license number 5805530902892) [50].

In this approach, the nutrient medium is tailored to meet the specific requirements of the bacterial strain. Following inoculation, the medium remains undisturbed. In cellulose production, during static conditions, aerating the medium in a well-ventilated area enhances production by ensuring adequate oxygen supply. Additionally, some researchers have noted that cellulose production from *Acetobacter xylinum* species may be increased by excluding light. Although the static culture method is widely used, its primary drawback is the extended fermentation period. Moreover, inadequate nutrient and oxygen distribution during fermentation can lead to uneven cellulose production, resulting in variations in thickness across the cellulose mat [47]. Hsieh and colleagues introduced a modified approach within the static culture method itself, involving intermittent feeding of the nutrient medium during cultivation. In this method, after a certain number of days of fermentation, a fresh batch of nutrient medium is introduced into the existing static culture medium. This new medium is poured directly onto the newly formed pellicle from the previous fermentation cycle. This process can be repeated multiple times until several layers of pellicles are formed. The researchers observed that this method enables continuous cellulose production, with an average of 0.02 g/day even after 30 days of cultivation, in contrast to the previous cultivation method where production ceases entirely [48]. Figure 2 depicts the BC production in static and agitated/shaking environments as each method offers distinct benefits.

#### Fed-Batch

In a fed-batch process, the operation begins as a batch process, but nutrients are later added either continuously or intermittently for a set duration without removing any culture fluid. This method keeps the substrate concentration at optimal levels for microorganism performance [49].

### 2.2. Agitated/Shaking Culture

Unlike the static method, in the dynamic method, the inoculated culture medium is placed in a rotary shaker throughout the fermentation or incubation period. The constant agitation of the culture enhances oxygen supply in the liquid medium containing the *A. xylinum* bacterial strain. However, this method results in cellulose formation in the shape of spheres, or it can be manufactured in other diverse shapes including fibrous suspensions, pellets, and irregular masses [47,48]. Therefore, this approach is more conducive to achieving higher cellulose production within a shorter timeframe compared to the static medium [47]. Table 1 simply shows the generic comparison of the static and agitated culture of bacterial cellulose production methods with their properties, nutrition type, production yield, etc.

#### Bioreactor Culture

To address the emergence of non-cellulose mutants in agitated culture and enhance the efficiency of BC production, various types of bioreactors have been utilized. These bioreactors have been found to yield significant amounts of BC. Different bioreactor configurations for BC cultures are distinguished by their operational methods, such as BC production under oxygen-enriched air, the utilization of rotating discs, or reliance on biofilm support [43].

## 3. Effect of Drying Characteristics of Bacterial Cellulose Aerogels on Their Thermal and Mechanical Behavior

**Figure 3:**
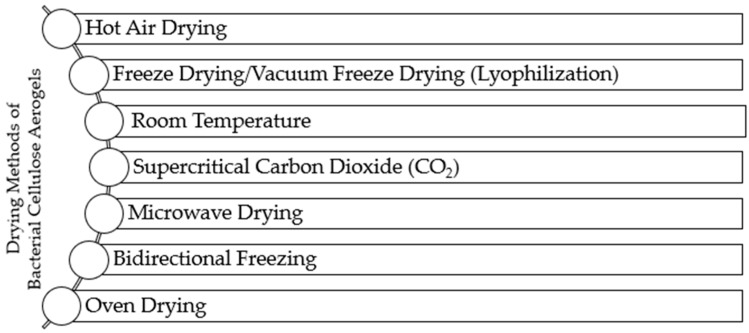
Most used drying methods are used for bacterial cellulose aerogels.

Bacterial cellulose and its composites can undergo various characterization methods, with the significance of these properties differing depending on the application. The rapid advancements in hydrogel research have placed hydrogels in scenarios that test their thermal and mechanical limits [24]. On that point, the drying methods of the BC aerogels are also important as well as its growing methods because drying can dramatically affect its characteristics, such as pores, specific surface area, morphology, crystallinity, absorbability, and, additionally, thermal and mechanical properties. In this section, the thermal and mechanical behavior of BC aerogels is discussed in detail. Figure 3 depicts the most used drying methods for bacterial cellulose aerogels.

### 3.1. Most Used Drying Methods

#### 3.1.1. Hot Air Drying

In this method, thermal energy is imparted to the product via hot air, facilitating moisture removal through the process of convection. A notable limitation of this technique is the comparatively extended duration required for effective drying. Furthermore, the direct application of heat can potentially compromise the quality of the product [56].

#### 3.1.2. Freeze-Drying

Freeze-drying is a dehydration process conducted at low temperatures. This technique involves freezing the material and subsequently removing its moisture content through the sublimation of ice. Freeze-drying is favored over conventional heat-drying and evaporation methods due to the superior quality of the resultant samples. The primary advantage of this method is its ability to preserve the shape of the sample, which is largely attributed to the low-temperature processing conditions [57].

#### 3.1.3. Room Temperature

Room temperature drying is a simple and cost-effective method for drying bacterial cellulose, involving exposure to ambient air until constant weight is achieved. Moisture loss is measured by the difference in mass before and after drying. This method can cause structural changes in porosity and density due to atmospheric pressure. Comparative studies show similar crystallinity between room temperature and freeze-dried samples (71% and 76%, respectively). Room temperature drying results in thinner films and higher fiber density compared to freeze-drying at −80 °C [58].

#### 3.1.4. Supercritical CO_2_ Drying

Supercritical CO_2_ drying, or critical point drying, removes moisture using CO_2_ at 74 bar and above 31 °C. In this supercritical state, CO_2_ penetrates samples effectively, making the process faster than other methods. CO_2_ can be recycled, enhancing energy efficiency and sustainability. Researchers have used this method to study bacterial cellulose drying, comparing it with freeze-drying and room temperature drying [59].

#### 3.1.5. Microwave Drying

Several researchers have employed microwave ovens, traditionally used for cooking, to dry bacterial cellulose. This technique, which is also prevalent in the food industry for drying vegetables and fruits, uses high-frequency electromagnetic waves in the range of 300 MHz to 300 GHz. It is regarded as an energy-efficient method. Microwave drying shortens processing time by converting the absorbed electromagnetic waves into heat, which facilitates the evaporation of moisture within the material [60]. Compared to hot air oven drying, microwave drying yields a smoother bacterial cellulose surface. This smoothness results from the even heat distribution throughout the structure due to water molecule vibrations. In contrast, hot air drying can cause surface wrinkles due to rapid water evaporation [47].

#### 3.1.6. Bidirectional Freezing

**Figure 4:**
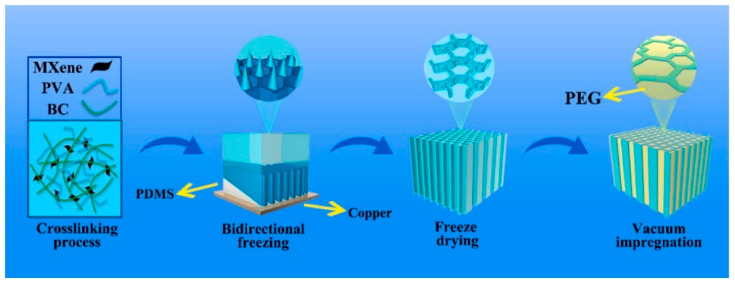
Diagram illustrating the preparation process of BPM aerogel and BG PCM [(BPM: BC/PVA/MXene) bacterial cellulose (BC)/polyvinyl alcohol (PVA)/(Ti_3_C_2_ MXene) [BG: BPM/PEG (polyethylene glycol), (PCM: phase change material)]. [Permission was approved by Elsevier, Food Hydrocolloids, under license number 5810801416550] [63].

Directional freezing involves freezing from one direction to produce clear ice. In a domestic freezer, this can be performed by placing water in an insulated container, allowing it to freeze from the top down. The container is removed before fully frozen to avoid mineral incorporation [61]. However, the bidirectional freezing technique organizes small constituents, including ceramic particles, platelets, and polymers, into a large-scale, single-domain porous lamellar structure measuring in the centimeter range. This structure is analogous to natural nacre, albeit lacking the “mortar” component [62]. The bidirectional freezing technique is an advanced method for creating anisotropic porous structures with specialized spatial arrangements. According to Zhu et al., they synthesized the aerogel via bidirectional freezing, incorporating PVA (polyvinyl alcohol) and BC as the matrix and Ti_3_C_2_ MXene as the filler for enhanced thermal conductivity. A wedge-shaped PDMS (Polydimethylsiloxane) structure induced temperature gradients in both vertical and horizontal directions, thereby regulating ice crystal formation during the freezing process. The microstructural characteristics and thermophysical properties of the aerogels were systematically investigated by varying the PDMS wedge angle from 0° to 30°. The resulting BC/PVA/MXene (BPM) aerogels demonstrated promising applications in photothermal harvesting and conversion, particularly in solar thermal systems. In Figure 4 is an illustration of the preparation process of BPM aerogel and BG PCM [(BPM/PEG (polyethylene glycol)) phase change material]. According to the study, anisotropic PCM composites made with a bidirectional freezing process have good thermal conductivity, photothermal conversion, and storage properties. This approach uses simultaneous temperature gradients in both horizontal and vertical directions, with the long-range aligned structure acting as a directional heat transport channel. Bidirectional aligned PCMs with temperature gradients of 0° and 20° exhibit high thermal conductivities of 0.716 W m^−1^ K^−1^ and 0.768 W m^−1^ K^−1^, which are 184% greater than PEG. They also show photothermal conversion efficiencies of 55.17% and 76.91 percent. Furthermore, the vertically oriented pore structure confines the PEG, resulting in form durability and a high enthalpy of 157.7 J/g [63].

#### 3.1.7. Oven Drying

The oven-drying method, a thermo-gravimetric process involving consistent temperature drying for a set duration, is commonly employed to assess moisture content in materials by comparing original and dried weights [64]. This method’s straightforward application makes it widely favored for drying bacterial cellulose. Florentina Sederaviciute et al. utilized this method to investigate the drying effects on the mechanical properties of cellulose films produced from kombucha-derived *G. bacter xylinus* in a tea medium. Following neutralization with distilled water and gentle tissue paper pressing, the cellulose films were dried at temperatures of 25 °C, 50 °C, and 75 °C. Results showed that higher temperatures led to quicker and more significant moisture loss, with losses recorded as 91.8% at 50 °C and 88.3% at 75 °C during the specified drying period [65].

In the rest of the sections, the effect of the drying methods of BC aerogels on thermal and mechanical behavior will be explained according to the composition of BC aerogels, including those produced with polymeric, inorganic, and other additives for each characterization.

### 3.2. Effect of Drying Methods of BC Aerogels on Thermal Behavior

#### 3.2.1. Effect of BC Aerogels Composed of Polymeric Additives on Thermal Properties

The significance of thermal analysis in hydrogel manufacturing is in its impact on processing, storage, and application. For instance, thermal analysis is crucial for identifying the optimal processing temperature to produce hydrogels with the desired properties. Moreover, the insights gained from thermal analysis ensure that the hydrogel is stored at suitable temperatures to prevent degradation and extend its shelf life [24]. As the drying methods are also crucial for BC aerogels, we aim to show the importance of drying methods; even though limited, there is some research on the different drying methods with different types of bacteria. Zhang et al. used the *Gluconacetobacter Xylinum* strain for the preparation of BC gelatinous membranes dried using three different methods, which are (BC1) hot air drying at 60 °C, (BC2) vacuum oven drying at 60 °C, and (BC3) vacuum freeze-drying at −50 °C. The BC gelatinous aerogel samples were characterized in SEM (scanning electron microscope) for the surface and a section; with an FT-IR (Fourier transform infrared spectrometer); and with X-ray diffraction for the crystallinity of BC films. Their results indicated that films dried using hot air displayed a uniform and dense surface. In contrast, films dried under vacuum exhibited cavities and cracks, while those subjected to vacuum freeze-drying revealed layered and porous structures. FT-IR spectra and X-ray analysis confirmed that vacuum freeze-drying weakened the hydrogen bonds between cellulose macromolecules and reduced the degree of crystallinity in bacterial cellulose films, though it had minimal impact on the crystal structure itself [32]. In general, compared to supercritical drying, the freeze-drying method offers several advantages: it is environmentally friendly, straightforward, and cost-effective. Additionally, the porous structure can be easily tailored by using water-soluble polyamic acid as a precursor. Fan et al.’s previous research demonstrated that polyimide (PI) aerogels prepared through freeze-drying exhibit a thermal conductivity of 53 mW m^−1^ K^−1^, attributed to the large pore sizes formed during the freezing process [66]. In the research conducted by Zang et al., a lightweight, bidirectional anisotropic aerogel composed of polyimide and bacterial cellulose (b-PI/BC) was developed using a bidirectional freezing technique. Bacterial cellulose, with its superfine nanofibrous structure, serves as a reinforcing nanofiller for PI aerogels. This inclusion prevents shrinkage and maintains the aerogel’s structural integrity, resulting in higher porosity and lower density. These characteristics enhance thermal insulation by reducing heat conduction. Compared to random and unidirectional freezing methods, the b-PI/BC aerogel produced through bidirectional freezing exhibits a well-aligned lamellar porous structure. This parallel lamellar configuration significantly reduces heat transfer perpendicular to the lamellae while facilitating in-plane heat diffusion, thus preventing heat localization. The b-PI/BC aerogel demonstrates an exceptionally low thermal conductivity of 23 mW m^−1^ K^−1^ in the radial direction (perpendicular to the lamellae) and nearly double that value (44 mW m^−1^ K^−1^) in the axial direction (parallel to the lamellae) [67]. Regarding the improvement of the thermal stability of BC, the researchers used two different composites, where one of them is with collagen and its three forms of collagen gel, collagen solution, and hydrolysed collagen with lyophilization and another is a mixture of BC powder in collagen solutions (collagen solution, collagen gel, and hydrolysed collagen) with, again, lyophilization. The findings indicated that BC functions as a thermal stabilizer for the collagen solution (CS) matrix, while it synergistically interacts with the collagen gel (CG) matrix, resulting in composites with enhanced properties. Furthermore, BC sheets impregnated with collagen demonstrated significantly improved thermal stability [68]. Vasconcellosa et al. also studied the effect of two different drying methods on the properties of bacterial cellulose aerogel, which were oven drying at 50 °C and freeze-drying. They only used the *Gluconacetobacter hansenii*-type bacteria. The two samples exhibited distinct visual characteristics: the oven-dried BC was transparent, while the freeze-dried BC appeared whitish. SEM images revealed that both samples had a similar interwoven structure. However, the freeze-dried material displayed higher porosity, whereas the oven-dried sample showed collapsed fibers, resulting in reduced film volume. Thermal stability analysis indicated that both films had comparable degradation profiles, with degradation beginning at 319 °C for the oven-dried sample and 325 °C for the freeze-dried sample. Both BC films exhibited similar crystallinity levels (85%), although their diffractograms showed different peaks. This suggests that the drying process altered the ratio of Iα/Iβ polymorphs in the films, a finding supported by FT-IR results [33]. 

There are certain limitations to the insulation properties of cellulose aerogels: (1) thermal conductivity is primarily influenced by solid and gas conduction (approximately 70%), and (2) gas conduction significantly diminishes when the average pore size is much smaller than the mean free path of gas molecules, a phenomenon known as the Knudsen effect. Low bulk density is advantageous in this context, as high porosity and small beam diameters inhibit the propagation of phonons in the delicate scaffold, thereby reducing the contribution of solid-phase radiation [69,70]. Due to the demand for novel thermal insulation materials and the need to enhance bacterial cellulose’s thermal properties, research in this area has grown despite some limitations. Traditional materials fall short in intelligent heat management, driving interest in new solutions. This study focused on bidirectional anisotropic polyimide/bacterial cellulose (b-PI/BC) aerogels, prepared via a bidirectional freezing technique. These aerogels demonstrated excellent structural formability, mechanical strength, and thermal insulation. Polyimide enhanced mechanical robustness, while bacterial cellulose prevented shrinkage, maintaining structural integrity and resulting in higher porosity and lower density. Consequently, the aerogels exhibited anisotropic thermal insulation with an ultra-low thermal conductivity of 23 mW m^−1^ K^−1^ radially and 44 mW m^−1^ K^−1^ axially, effectively reducing radial heat transfer and facilitating in-plane heat diffusion, making them ideal for complex thermal insulation applications [67].

Another study showed that a thermal conductivity as low as 13 mW/(K·m) was recorded for native pellicle-based aerogels by using *Gluconacetobacter hansenii* bacteria dried in their original state with minimal additional processing using the super critical CO_2_ drying method. Utilizing waste from the beer-brewing industry as a growth medium for the pellicle preserved the cellulose yield achieved with conventional Hestrin–Schramm media, enhancing both the cost-effectiveness and sustainability of our product for a reduction in energy consumption in building applications [71]. 

#### 3.2.2. Effect of BC Aerogels Composed of Inorganic and Different Types of Additives on Thermal Properties

Despite BC aerogels having strong thermal insulation performance due to their dense inner porous network, they are easily ignited and burned out, restricting their potential uses as domestic appliances or materials for construction. Improving BC aerogels’ resistance to flames has turned into a major focus of numerous fascinating scientific projects [72]. Adding other agents, such as graphene, halogens, clay, phosphorus, nitrogen, organic polymers [73,74], and metallic nanoparticles, by in situ forming or doping inside the BC network is a practical strategy [75]. Collaborating additives could take the form of a dense protective layer on the surface and segregate oxygen to achieve a flame-retardant effect. Researchers Wang et al. developed a flame retardant and heat-insulating composite aerogel by incorporating zinc borate (ZnB) particles into bacterial cellulose (BC) using an ultrasound-assisted deposition process followed by freeze-drying. The in-situ formation of ZnB particles facilitated the separation of individual fibers from fiber bundles within BC, preserving its homogeneous porous structure. This modification enhanced the thermal stability of the aerogel by diminishing the convective heat transfer. The composite aerogel demonstrated outstanding flame retardancy, with a heat release capacity of merely 8 J g^−1^ K^−1^. The suggested mechanism involves the dehydration of ZnB particles, which lowers the surface temperature by releasing bound water and concurrently produces metallic oxides (ZnO and B_2_O_3_), which inhibit heat propagation and isolate flammable fibrils within the combustion zone [76]. Sai et al. modified the surface of bacterial cellulose aerogel (BCA) using a trimethylsilylation reaction with trimethylchlorosilane (TMCS) in the liquid phase followed by freeze-drying. Compared to the original cellulose aerogel, thermogravimetric analysis (TGA) revealed notable differences in the thermal properties of the trimethylsilylated cellulose aerogels. It is well established that the thermal decomposition of cellulose can be accelerated by modification with organic acids and acyl chlorides (such as acetic acid, dodecanoic acid, p-toluenesulfonyl chloride, oleic acid, and stearic acid). In this study, the decomposition temperature of BCAs showed a slight increase after trimethylsilylation, with higher degrees of substitution (DS) resulting in higher decomposition temperatures (shifting from 370 °C to 380 °C). This indicates that TMCS has a stabilizing effect on bacterial cellulose when subjected to lyophilization. While silica is known to raise the decomposition temperature of cellulose, TMCS, which contains silicon, also forms Si–O bonds upon reacting with cellulose. Therefore, the presence of silicon and Si–O bonds likely contributes significantly to the improved thermal stability of the modified cellulose [77]. 

**Figure 5:**
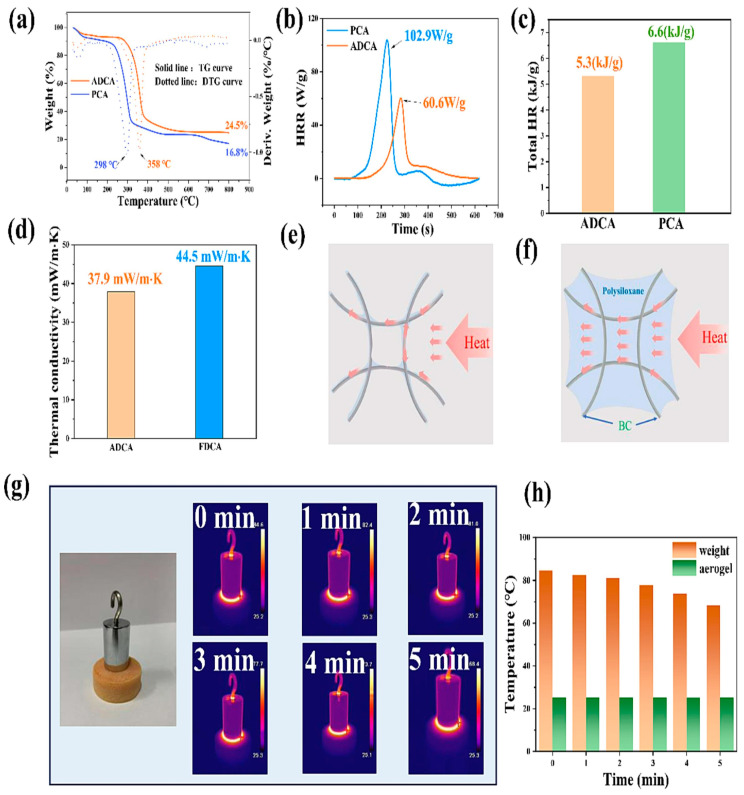
(**a**) Thermogravimetric analysis of PCA and ACDA. (**b**) Heat release rate profiles for PCA and ACDA. (**c**) Total heat release data for PCA and ACDA. (**d**) Thermal conductivity measurements for ADCA and FDCA. (**e**) Diagram illustrating the heat conduction mechanism in ADCA. (**f**) Another diagram depicting the heat conduction mechanism in ADCA. (**g**) Infrared thermal images of ADCA. (**h**) Temperature changes in weight and ADCA aerogel over a 5 min period (permission was approved by Elsevier, Food Hydrocolloids, under license number 5806700171839) [78].

Besides the freeze-drying method, some of the studies have also shown the effect of the ambient/room temperature drying method by using the purchased 0.8% bacterial cellulose with γ-aminopropyl triethioxysilane (APTES) linked by the freeze-linking technique. Aerogels produced through atmospheric pressure drying are cost-effective to manufacture and can be redried through solvent exchange even after being used in aqueous environments. As per the research results, during the solvent exchange process, the loss of some polysiloxane leads to defects in the aerogel. Aerogels prepared by atmospheric pressure drying are cost-effective and re-dryable after aqueous use. Thermogravimetric analysis in Figure 5 shows that PCA has a maximum mass reduction at 298 °C, while ADCA’s (atmospheric drying cellulose aerogel) occurs at 358 °C due to its double crosslinked network. PCA’s (pure cellulose aerogel without APTES) carbon residue is 16.8%, compared to ADCA’s 24.5% due to Si. Heat release rate tests indicated that PCA’s maximum rate is 102.9 W/g, while ADCA’s is 60.6 W/g, with the total heat release of ADCA at 5.3 kJ/g versus PCA’s 6.6 kJ/g due to N and Si’s flame-retardant effect. Thermal conductivity for FDCA (freeze-drying cellulose aerogel) is 44.5 mW/m·K and for ADCA is 37.9 mW/m·K. FDCA’s dense structure aids solid heat conduction, whereas ADCA’s structural defects from APTES removal reduce it. Infrared imaging showed that heat dissipates through air, not ADCA. Freeze-drying forms the aerogel skeleton but creates dense structures; APTES removal during solvent replacement reduces solid heat conduction, suggesting a way to enhance aerogel insulation properties. This not only lowers its thermal conductivity but also increases its specific surface area. Additionally, the aerogel’s water resistance, thermal performance, and dye adsorption capabilities are significantly enhanced [78].

Bacterial cellulose aerogels exhibit low thermal conductivity, similar to loose-fill cellulose. For commercialization, identifying optimal cultivation conditions—considering the bacterial strain, growth medium composition, additive types, and cultivation techniques—is crucial. These conditions should facilitate the formation of largely isotropic cellulose networks with nanopores having a narrow size distribution and maximum pore diameters not exceeding 70 nm. Such finely tuned structures can potentially serve as super-insulating materials.

**Figure 6:**
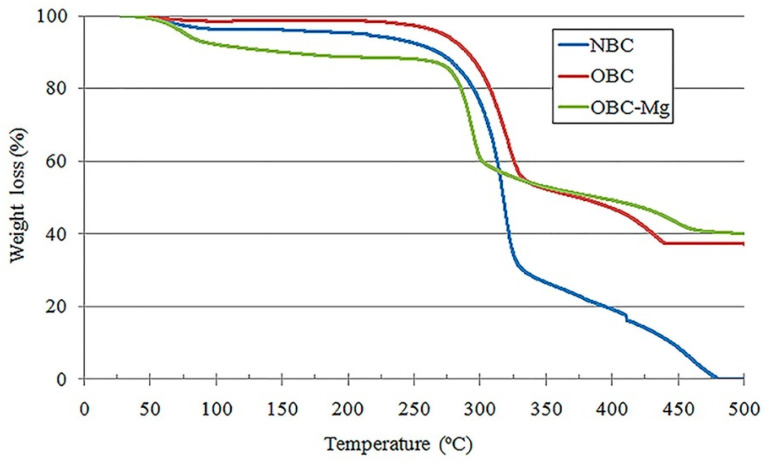
A thermogravimetric analysis was performed on BC aerogels made from native bacterial cellulose (NBC) and TEMPO-oxidized BC with an oxidation degree (OD) of 1.44%, with and without 20 mM Mg^2+^. [The article “Production of Bacterial Cellulose Aerogels with Improved Physico-Mechanical Properties and Antibacterial Effect” is an open-access article distributed under the terms of the Creative Commons Attribution License (CC BY). The use, distribution, or reproduction in other forums is permitted, provided the original author(s) and the copyright owner(s) are credited and that the original publication in this journal is cited, in accordance with accepted academic practice] [81].

Revin et al. optimized the fabrication of robust, thermally efficient aerogels derived from BC synthesized by *Komagataeibacter sucrofermentans* H-110 and freeze-dried. Aerogels made from TEMPO-oxidized BC (OBC) exhibited enhanced strength and reduced shrinkage compared to those made from native BC (NBC). The addition of 20–40 mM Mg^2+^ further increased the aerogel strength by 4.9 times, minimized pore size, and decreased thermal conductivity from 0.036 to 0.0176 W/(m·K). Additionally, aerogels incorporating sodium fusidate demonstrated significant antibacterial activity against Staphylococcus aureus, making them suitable for applications in tissue engineering and wound dressing. The temperature stability of BC aerogels is crucial for high-temperature applications. In the study, a thermogravimetric analysis was conducted on BC aerogels made from native bacterial cellulose (NBC) and TEMPO-oxidized BC with an oxidation degree (OD) of 1.44%, with or without 20 mM Mg^2+^ (Figure 6). The NBC aerogel exhibited initial decomposition between 275 °C and 330 °C, with a weight loss starting at 57 °C and reaching 7.5% at 250 °C, attributed to the evaporation of water related to bacterial cellulose [79]. The thermal decomposition of the NBC aerogel began at 275 °C, resulting in a 70% mass loss by 330 °C, consistent with previous findings on BC film thermal stability [80]. The OBC and OBC-Mg aerogels showed main mass losses beginning at 275 °C, reaching 43% and 39% by 330 °C and 300 °C, respectively. At 500 °C, the mass loss for OBC and OBC-Mg aerogels was 63% and 60%, respectively, while the NBC aerogel lost 99% of its mass. These results indicate that TEMPO oxidation does not alter the thermal stability of BC, though the nature of thermal degradation varies with BC oxidation and the presence of Mg^2+^ [81].

### 3.3. Effect of Drying Methods of BC Aerogels on Mechanical Behavior

#### 3.3.1. Effect of BC Aerogels Composed of Polymeric Additives on Mechanical Behavior

The mechanical qualities of bacterial cellulose are principally determined by its chemical composition and structural features. Increased fiber alignment and crystallinity result in a stronger material that can withstand more mechanical stress. Because crystallinity varies depending on the source and production processes, the mechanical characteristics of bacterial cellulose can differ. Post-chemical treatments and ex situ alterations can also improve or degrade certain qualities, depending on the chemicals used in the composite synthesis. As previously noted, as BC properties are influenced by the production process, consequently, so are its applications. The impact of various cultivation methods (static or agitated; batch or fed-batch processes), different bacterial strains, and types of culture media (complex or industrial waste) on BC properties has been extensively studied. Additionally, downstream processing treatments can significantly affect the final characteristics of BC. In particular, the effects of different drying processes on BC properties continue to be a subject of ongoing research [33,82]. Therefore, BC films were produced by *G. hansenii* using static cultivation, followed by a purification step and subsequent drying through two different methods: oven drying and freeze-drying. The diverse applications and biological functions of BC membranes are attributed to their unique morphology, with the intertwining fibers in the films providing significant mechanical strength and water absorption capacity, as highlighted in the studies by Vasconcellos et al. [33]. Their findings, along with those of Zeng et al., who evaluated three different drying processes—room temperature drying, freeze-drying, and supercritical CO_2_ drying—revealed that the drying method impacts mechanical properties such as penetration depth, hardness, and water absorption capacity, with each method enhancing specific characteristics [58]. In another study, the effect of different cultivation methods and freeze-drying with different freezing modes on the physical and mechanical properties was examined by using the strain of *Gluconacetobacter sucrofermentans* H-110 bacterial cellulose aerogels. The bacterial cellulose hydrogel samples underwent freezing in a controlled manner, utilizing foil containers sized at 90 mm × 60 mm × 25 mm (length × width × height) in a single stage freezing process. Attempts at rapid freezing in liquid nitrogen or multistage cooling did not produce aerogels with satisfactory strength characteristics. To ensure a uniform hydrogel, the bacterial cellulose gel film was subjected to grinding. By adjusting the concentration of bacterial cellulose in the hydrogels, the density, porosity, and average pore size of bacterial cellulose-based aerogels can be tailored. This allows for easy manipulation of aerogel properties during the initial processing of the bacterial cellulose. The mechanical grinding of bacterial cellulose was complemented with ultrasonic treatment, a widely used technique for polymer breakdown. This disintegration method does not modify the chemical composition of the polymers but reduces their molecular weight effectively [80]. In addition to the invaluable insights provided by thermal analysis techniques, mechanical analysis techniques also offer a wealth of crucial information. Commonly employed methods include compression testing, tensile testing, and dynamic mechanical analysis (DMA). Table 2 shows the effect of different drying methods on the thermal and mechanical properties of bacterial cellulose aerogel for different applications. Some studies show comparable methods, which are freeze-drying and traditional drying methods regarding the process speed, cost, and retention of the sample’s properties. Some studies show the effect of freeze-drying on the properties of the native BC aerogel compared to modified BC aerogel.

#### 3.3.2. Effect of BC Aerogels Composed of Inorganic and Different Types of Additives on Mechanical Properties

Cellulose aerogels can be obtained from aqueous dispersions of nanocellulose fibers (CNF), nanocellulose crystals (CNC), and bacterial cellulose (BC) [83]. Although CNC and CNF are renewable, their extraction processes are environmentally detrimental, requiring hazardous chemicals and significant energy consumption. BC, produced by microorganisms, is an eco-friendlier alternative. However, despite the high theoretical strength of these nanofibers, cellulose aerogels suffer from poor mechanical properties due to weak hydrogen bonding and physical entanglement among the fibers. This results in collapse when absorbing water and deformation under pressure, limiting practical applications [43]. Developing cellulose aerogels with robust mechanical properties, including high elasticity and fatigue resistance, is essential for their effective use in thermal insulation, oil contamination adsorption, and high-performance sensors [84,85]. Generally, cellulose aerogels can be chemically changed, crosslinked, or mixed with other functional materials to increase their elasticity. Crosslinking agents or functional modifiers can increase both the mechanical and hydrophobic properties of cellulose aerogels [86]. This increase is mostly owing to cellulose’s hydroxyl groups, which serve as target locations for the majority of modification and crosslinking techniques [87]. Studies have focused on improving the resilience of BC aerogels generated for oil/water separation by thermochemical vapor deposition (CVD) mediated by 1,2,3,4-butanetetracarboxylic acid (BTCA). BTCA performs two functions: esterification with BC and accelerating CVD after freeze-drying. The resulting aerogel recovers rapidly following compression, with elastic recovery surpassing 90% at a maximum deformation of 80%. It also has high fatigue resistance, with over 80% elastic deformation after 50 cycles. This highly elastic and hydrophobic aerogel is perfect for oil absorption and desorption using simple mechanical squeezing. Its adsorption capabilities for n-hexane and dichloroethane persist at 87% and 81% after 50 cycles, respectively, suggesting high reusability [88]. Another study outlines an eco-friendly, one-pot synthesis method for creating a versatile bacterial cellulose/γ-(2,3-epoxypropoxy) propytrimethoxysilane composite aerogel (BK aerogel) following freeze-drying. This composite aerogel demonstrates exceptional elasticity, retaining up to 96.4% of its original form after being compressed at an 80% strain. Furthermore, it exhibits notable fatigue resistance, achieving an elastic recovery of 87.8% after 50 compression cycles at the same strain level. The BK aerogel can effectively absorb cationic dyes when immersed in water and can be reused multiple times without experiencing degradation. The aerogel’s richness in hydroxyl and epoxy groups allows for extensive surface chemical modifications. For example, through hydrophobic modification, the aerogel can be utilized for oil–water separation and is capable of being reused through mechanical squeezing [89].

**Figure 7:**
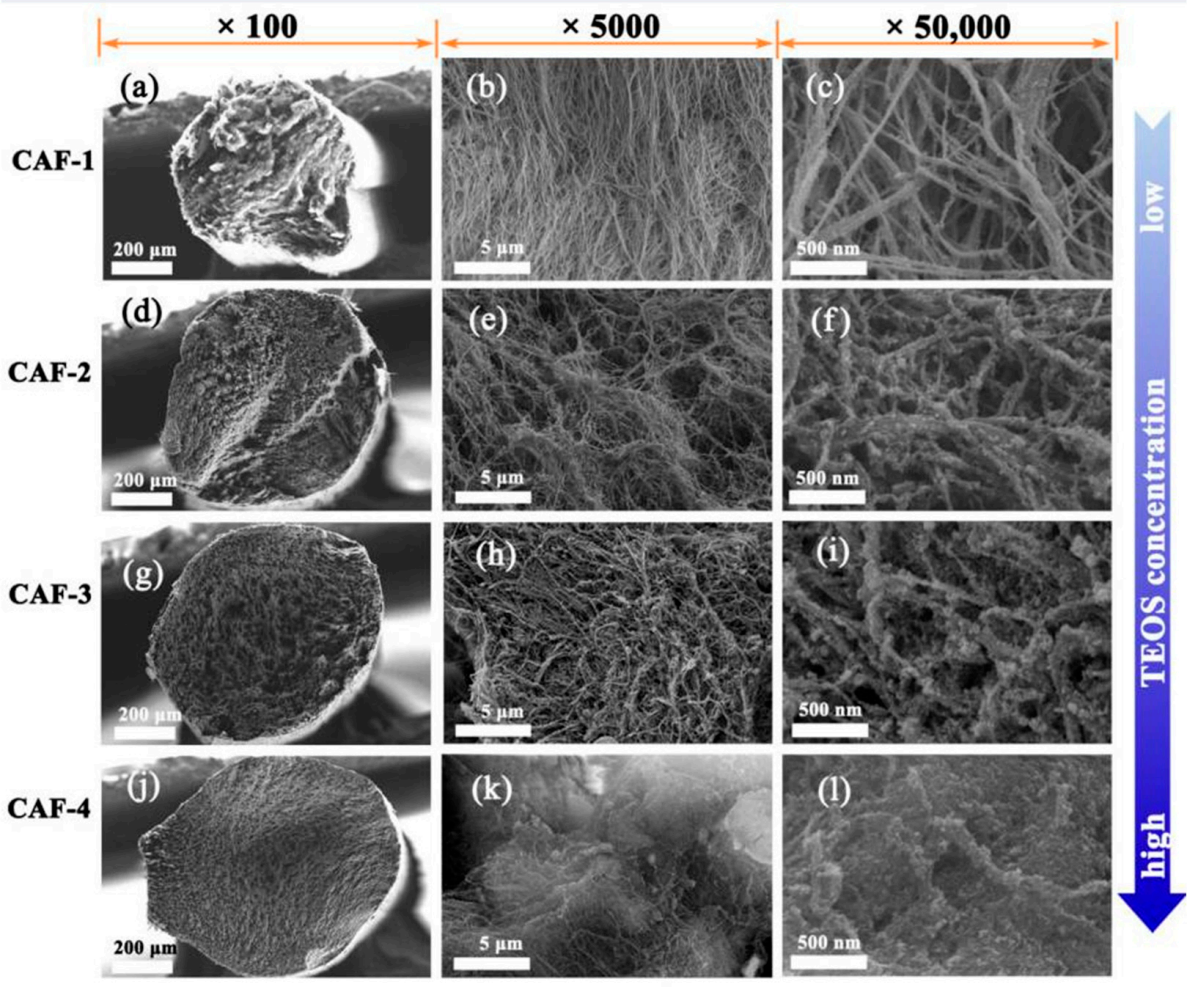
The SEM images of CAF-1 (**a**–**c**), CAF-2 (**d**–**f**), CAF-3 (**g**–**i**), and CAF-4 (**j**–**l**) are shown at various magnifications (CAFs: Silica–BC composite aerogel fibers (CAFs)) [Licensee MDPI, Basel, Switzerland. The article “Robust Silica-Bacterial Cellulose Composite Aerogel Fibers for Thermal Insulation Textile” is an open-access article distributed under the terms and conditions of the Creative Commons Attribution (CC BY) license (https://creativecommons.org/licenses/by/4.0/)] [90].

To enhance the mechanical properties of aerogel blocks or films, high-performance fibrous silica-BC composite aerogels were synthesized using freeze-drying in one of the studies, andthis structure has been shown to possess significantly superior mechanical properties. Silica sol was infused into a BC hydrogel matrix, which was then cut and reshaped to form a composite wet gel fiber with a nanoscale interpenetrating network. This process increased the tensile strength of the aerogel fibers to 5.4 MPa by significantly increasing the quantity of BC nanofibers per unit volume. The composite aerogel fibers exhibited a high specific surface area (up to 606.9 m^2^/g), low density (less than 0.164 g/cm^3^), and excellent hydrophobicity. SEM images in Figure 7 revealed that at low precursor concentrations, the aerogel fibers were primarily composed of BC nanofiber aggregates with minimal silica attachment. At higher concentrations, a silica gel skeleton formed within the BC network, creating a more compact structure. The diameter of the samples, approximately 0.7 mm, indicated minimal shrinkage during drying. Ensuring an adequate concentration of TEOS (Tetraethoxysilane) precursors was crucial for constructing a robust gel skeleton to maintain structural integrity during the drying process [90].

**Table 2:** Some research on effects of drying methods shows thermal, mechanical, and other properties of BC aerogels.

BC Aerogels	Effect of Drying Methods on Thermal & Mechanical Behavior of BC Aerogels	Drying Methods	Applications	Ref.
Gluconacetobacter xylinus BC membrane	The material’s ability to swell is much more decreased with evaporation drying than freeze-drying. Gas permeability of freeze-dried membranes is higher than evaporation-dried membranes. The Young’s modulus of polymer membranes varies depending on the bacterial strain used.	Freeze-drying, Evaporation drying	Wet wound dressing	[91]
Fermented coffee kombucha (CK) BC aerogels by Gluconacetobacter	Oven-dried BCs exhibited the highest tensile stress at break, measuring 24.67 ± 4.40 MPa. All BCs decomposed between 230 and 400 °C regardless of drying conditions.	Oven drying (OD), freeze-drying, vacuum oven drying, and Büchner funnel vacuum drying (BFVD).	Distinctive applications in various industries	[92]
Gluconacetobacter sucrofermentans H-110 BC aerogels	With an aerogel density of 22.8 kg/m^3^, the modulus of elasticity at 80% compression was 0.1 MPa. Lower aerogel density resulted in larger pore sizes (20 to 1000 μm) and reduced modulus of elasticity.	Freeze-drying	Heat- and sound-insulating materials	[80]
Comparing of native BC aerogel and TEMPO ((2,2,6,6-tetramethylpiperidin-1-yl)oxyl)) oxidized BC aerogels	Aerogels synthesized from oxidized BC demonstrate increased durability and reduced shrinkage relative to those produced from native BC through freeze-drying. Additionally, TEMPO-mediated oxidation of BC, in conjunction with Mg^2+^ addition, yields aerogels with substantially improved mechanical strength and a more uniform microporous architecture.	Freeze-drying	Biomedical applications	[81]
Natively produced BC pellicles by Gluconacetobacter hansenii	Native cellulose aerogels exhibited a very low thermal conductivity of 13 mW/(K·m). Mechanical and thermogravimetric analysis demonstrated the potential of BC aerogels with added carboxymethyl cellulose for building insulation. These aerogels could enhance thermal insulation and add fireproofing properties in multilayer insulation blankets.	Super critical carbon dioxide drying	Building envelope applications	[71]
Bacterial cellulose films	Microwave drying of films is 95% faster than air convection drying. The structure, color, and mechanical properties of BC films dried by microwave and air convection heating were nearly identical. However, microwave-dried films had slightly lower crystallinity and higher swelling. Elongation of samples with air convection drying was higher than microwave oven drying at break.	Microwave oven and air convection heating drying	Food packaging and edible film	[28]
Gelatinous Bacterial Cellulose Film by Gluconacetobacter Xylinum	The mechanical properties of BC films prepared by vacuum freeze-drying were inferior to those prepared by other methods; hot air drying showed the best results on mechanical properties.	Hot air drying, vacuum drying and vacuum freeze-drying	-	[32]
Producing of Komagataeibacter hansenii 23769 and Herman Schermann BC aerogels	Oven-dried samples’ Young’s modulus, tensile strength, and lower strain are higher than those of the freeze-dried ones. Oven-dried BC had higher crystallinity, LOI (lateral order index), and lower porosity with narrower fiber diameter and distribution than freeze-dried BC, regardless of bacterial strains.	Oven and freeze-drying	Anodic applications	[29]
Kombucha bacterial cellulose (KBC)	The microwave drying method showed a lower activation energy (131.70 Wg^−1^), higher moisture diffusivity (48.27 × 10^−11^ m^2^s^−1^), and greater tensile strength (59.45 MPa). It was more efficient due to faster drying, higher rehydration ratio, and increased tensile strength. In contrast, room temperature drying was better for water affinity.	Microwave drying (180–900 W), hot air oven drying (30–70 °C), and shade drying (25 °C).	Various applications	[93]
Dehydration of wet bacterial cellulose (BC) from OPF (oil palm frond) juice	Thermal analysis using TGA and DSC showed that hot-pressed BC had higher thermal resistivity but lower thermal stability than freeze-dried BC.	Hot-pressed and freeze-dried	-	[94]
BC aerogel from fruit waste by kombucha fermentation	The alterations resulted in significant improvements in aerogel mechanical properties, with rebound values exceeding 90%. Derived from three raw materials, the aerogels display low density, high porosity, and reduced thermal conductivity, suggesting suitability for insulation applications.	Two directional freeze-drying	Oil–water separation and thermal insulation	[95]

## 4. Conclusions & Future Perspectives

Although most studies indicate that the production method and bacterial strain used in producing BC aerogels are primary factors influencing their thermal and mechanical properties, it has also been observed that the final drying method significantly impacts these properties. Due to the high production costs associated with bacterial cellulose, drying methods that maintain the properties of BC aerogels while being cost-effective are preferred. As per the given research in the review, the thermal stability of BC aerogels, influenced by various treatments and additives, is crucial for high-temperature applications. The studies on the thermal behavior of BC aerogels showed that freeze-drying methods have improved the BC aerogels’ porous structure and decreased the thermal conductivity; moreover, depending on the type of composite structure used, it is environmentally friendly and low cost compared to supercritical CO_2_ drying. However, the studies on the mechanical behavior of BC aerogels showed that the mechanical qualities of BC are determined by its chemical composition and structural characteristics, such as fiber alignment and crystallinability. These properties, which differ depending on the source and manufacturing technique, determine the material’s capacity to sustain mechanical stress. Ex situ modifications and post-chemical treatments can both influence BC characteristics. Various cultivation methods, bacterial strains, and types of culture media have a considerable impact on BC characteristics. Furthermore, downstream processing techniques, notably varied drying methods, play an important impact in shaping the final properties of BC. According to studies, the drying procedure that is the most used is freeze-drying, which preserves the desirable porous structure and influences the final mechanical properties of the material, and it has an impact on qualities including penetration depth, hardness, and water absorption capacity. Despite ongoing studies on the effects of drying methods on bacterial cellulose aerogels, the research is still insufficient and opens new avenues for further investigation. Recent studies reveal that in primary drying methods like freeze-drying, not only the drying process itself but also the pre-drying freezing speed significantly affects the bacterial cellulose samples [96,97,98]. To realize the entire potential of this ecological material, detailed investigations on drying processes, cost reductions in BC manufacturing, improved structural and functional qualities, and the exploration of new solvents and potential uses are all required.

## Figures and Tables

**Table 1:** Comparison of static and agitated culture of bacterial cellulose growth.

Static Culture		Agitated Culture (Stirred Condition)	Ref.
1	3D Interconnected network-like films	Pointed, uneven sphere-like cellulose particles (SCPs)	[51]
2	Carbon and air supply	Stays completely dispersed within the culture medium	[52]
3	Enhanced genetic robustness	Commercial high yield	[53]
4	Higher Young’s modulus	Very low degree of polymerization	[37]
5	Established geometrics	Lower level of crystallinity	[54]
6	Significant water retention capability	Costly production	[55]
7	Strong wet tensile strength	-	[56]

## Data Availability

No new data were created or analyzed in this study. Data sharing is not applicable to this article.
